# The Prevalence and Factors Associated with Prophylactic Antibiotic Use during Delivery: A Hospital-Based Retrospective Study in Palembang, Indonesia

**DOI:** 10.3390/antibiotics10081004

**Published:** 2021-08-19

**Authors:** Ariesti Karmila, Mohammad Zulkarnain, Abarham Martadiansyah, Putri Mirani, Nuswil Bernolian, Joseph C. Gardiner, Lixin Zhang

**Affiliations:** 1 Department of Epidemiology and Biostatistics, College of Human Medicine, Michigan State University, East Lansing, MI 48824, USA; karmilaa@msu.edu (A.K.); gardine3@msu.edu (J.C.G.); 2 Department of Child Health, Faculty of Medicine, University of Sriwijaya, Palembang 30126, Indonesia; 3 Department of Public Health, Faculty of Medicine, University of Sriwijaya, Palembang 30126, Indonesia; mzulkarnian@unsri.ac.id; 4 Department of Obstetrics and Gynecology, Faculty of Medicine, University of Sriwijaya, Palembang 30126, Indonesia; abarhammartadiansyah@fk.unsri.ac.id (A.M.); putrimirani@fk.unsri.ac.id (P.M.); nuswilbernolian@fk.unsri.ac.id (N.B.)

**Keywords:** prophylactic antibiotics, delivery, low- and middle-income countries, adherence

## Abstract

Prophylactic antibiotic usage during delivery is a common practice worldwide, especially in low- to middle-income countries. Guidelines have been published to reduce antibiotic overuse; however, data describing the use of prophylactic antibiotics and clinician adherence to guidelines in low- to middle-income countries remain limited. This study aimed to describe the prevalence of prophylactic antibiotic use, factors associated with its use, and clinician adherence to guidelines. A retrospective review was conducted for all deliveries from 1 January 2016 to 31 December 2018 at a tertiary level hospital in Indonesia. The prevalence of prophylactic antibiotic use during delivery was 47.1%. Maternal education level, Ob/Gyn specialist-led delivery, a history of multiple abortions, C-section, premature membrane rupture, and antepartum hemorrhage were independently associated with prophylactic antibiotic use. Clinician adherence to the guidelines was 68.9%. Adherence to guidelines was the lowest in conditions where the patient had only one indication for prophylactic antibiotics (aOR 0.36, 95% CI 0.24–0.54). The findings showed that the prevalence of prophylactic antibiotic use during delivery was moderate to high. Adherence to local guidelines was moderate. Updating the local prescribing guidelines may improve clinician adherence.

## 1. Introduction

Bacterial infection during labor and delivery is one of the leading causes of maternal and neonatal mortality worldwide, accounting for about one-tenth of the global burden of maternal and neonatal deaths [1,2]. While the number of deaths from these infections has decreased considerably in high-income settings, the situation has not improved in many resource-limited settings [3,4,5,6]. In Indonesia, serious bacterial infections are responsible for about 600,000 newborn deaths every year. Neonatal sepsis is a major cause of neonatal mortality and accounts for 13 per cent of newborn deaths [7]. Infection is also one of the three leading causes of maternal death [8,9].

Reduction of bacterial infections is typically attempted by the prescription of prophylactic antibiotics during labor and delivery as a routine practice. Studies have shown that the use of antibiotics has reduced maternal infections and has improved neonatal outcomes. The incidence of neonatal sepsis has significantly declined since the introduction of guidelines by the Centers for Disease Control and Prevention (CDC) for the prevention of perinatal Group B Streptococcus (GBS) [10,11]. The benefit of prophylactic antibiotics in reducing infection incidence in women who have undergone a C-section has also been proven [12]. Prophylactic antibiotic use for the premature rupture of membranes (PROM) has also been associated with a reduction in neonatal infection [13].

These significant benefits of antibiotic use during labor and delivery have inevitably led to an increased use of antibiotics worldwide [14,15]. Even in the US, at some centers, the use of antimicrobials have more than doubled compared to the era before IAP introduction [15]. Studies in developing countries have reported that the proportion of deliveries that received antibiotics could reach up to 90% [16,17]. In Indonesia, study on prophylactic antibiotic use during delivery is very limited [18]. This situation immediately raised concern among experts, as previous studies had suggested that antibiotic exposure during labor and delivery may increase the risk of various adverse events to both the mother and newborn, including antibiotic-resistant bacterial infection, maternal and infant microbiome alteration, long-term functional impairment in children, and maternal anaphylaxis reaction [19,20,21,22]. Therefore, many countries, together with their professional organizations of physicians, have published guidelines that specify the recommended conditions for the administration of prophylactic antibiotics. These recommendations are supported by strong evidence on the impact and prevention of inappropriate use [14,23,24]. However, due to disparities in healthcare facilities, the guidelines for prophylactic antibiotic use in labor and delivery and the extent to which practitioners adopt these guidelines vary across countries, especially between high-income and low- to middle-income countries [14,18]. Despite the evidence that antibiotic use and inappropriate antibiotic prescriptions tend to be higher in low-income countries than in high-income countries, the practices of prophylactic antibiotic administration during delivery in low- to middle-income countries are not well characterized [25,26]. 

Antibiotic consumption (including inappropriate usage) is the major cause of antimicrobial resistance. Therefore, more data on antibiotic use practices from low-income countries, including prophylactic antibiotic practices during delivery, are essential for guiding and controlling the overuse and misuse of antibiotics to mitigate the development of antimicrobial resistance. This study utilized data on antibiotic use during delivery from three consecutive years at a tertiary-level referral hospital in Indonesia. The aims were to describe the prevalence of prophylactic antibiotic use during delivery, the significant factors that were associated with prophylactic antibiotic use, and clinician adherence to local guidelines. 

## 2. Results

From a total of 3957 recorded deliveries in the hospital during 2016–2018, the medical records of 3657 (92.4%) patients were retrieved: a total of 1087 of 1202 (90.4%) were from 2016, 1227 of 1338 (91.7%) were from 2017, and 1343 of 1417 (94.8%) were from 2018. Overall, the mean age of women who underwent delivery at this hospital was 29.9 years; and most of them were in the group age 17–35 (78.1%). Among the mothers, most were residents of Palembang city (63.8%) and most were high school graduates (72.9%). The gestational age of most deliveries was ≥37 weeks (77.1%), the parity was <5 (98.1%), previous abortions were ≤1 (97.3%), 97.6% had singleton births, 55.3% had vaginal delivery, and most (85.2%) were assisted by obstetrics and gynecology (Ob/Gyn) residents (Table 1). Across the calendar year, the maternal age group, place of residency, parity, multiple births, maternal leukocyte count, cases with antepartum hemorrhage, and foul-smelling amniotic fluid showed similar proportions. However, a significant difference was noted for the mother’s education level, birth attendant, number of previous abortions, gestational age, mode of delivery, maternal fever, and PROM cases.

### 2.1. Prevalence of Prophylactic Antibiotic Usage

**Figure 1:**
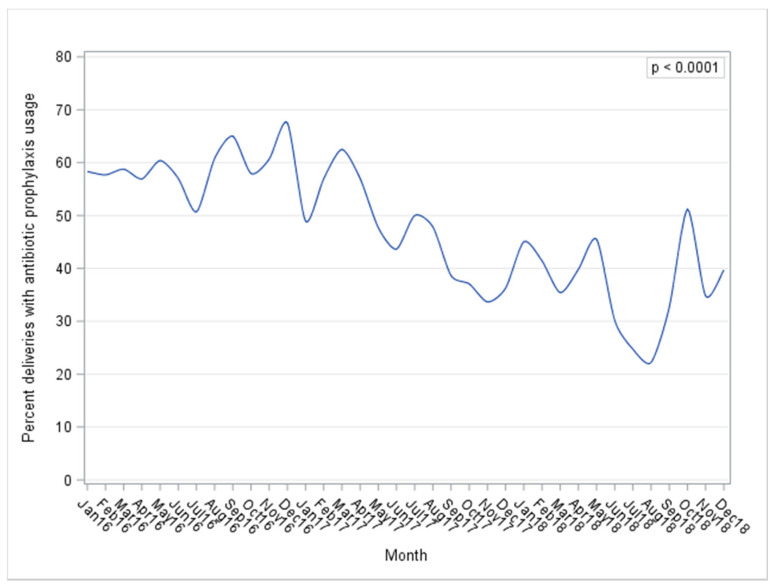
Percentage of deliveries with prophylactic antibiotic use during 2016–2018.

Of the 3657 deliveries, 2730 (74.7%) were given antibiotics during delivery. Of the given antibiotics, sixty-three percent were categorized as prophylaxis, which accounts for 47.1% of all deliveries. The prevalence of prophylactic antibiotic use was 63.6% for C-Section deliveries and 33.7% for vaginal deliveries. A marginal increase occurred at the end of 2018, but the proportion of women receiving prophylactic antibiotics decreased overall during the study, from 59.2% in 2016 to 46.2% in 2017 and to 38.1% in 2018 (Figure 1). The proportion decreased for both vaginal deliveries and C-sections. For vaginal deliveries, the proportion of prophylactic antibiotic use decreased from 42.4% to 33.2% and then to 24.4%. For C-sections, the proportion of antibiotic use also decreased, decreasing from 79.8% to 65.8 and then to 50%. Tests for linear trends over the years, which were based on Poisson regression, detected significant declining rates in prophylactic antibiotic use (*p* < 0.0001). 

Among the conditions where prophylactic antibiotics were recommended, the most common condition was C-section, followed by PROM, and then preterm (gestational age < 37 weeks) deliveries (Table 2). During the study period, ampicillin was the most commonly used antibiotic during delivery (64.2%), which was followed by ceftriaxone (34.2%).

The univariate analysis revealed significant associations of maternal educational level, place of residence, birth attendant, frequency of previous abortion, gestational age, mode of delivery, PROM, and antepartum hemorrhage with prophylactic antibiotic use. After adjustment, the associations between prophylactic antibiotic use and place of residence and gestational age lost their statistical significance. However, the associations between prophylactic antibiotic use and maternal education level, birth attendant, frequency of previous abortions, mode of delivery, PROM, and antepartum hemorrhage persisted (Table 2).

The factors relating to the choice of using ampicillin and ceftriaxone, the most frequently used prophylactic antibiotics at the study site, were also analyzed. The use of ceftriaxone was more likely to be suggested by Ob/Gyns than by residents in training (aOR 1.57, 95% CI 1.16–2.14). Patients with C-sections with leukocyte counts > 15,000/mm^3^, with an intrapartum temperature >38 °C, and with foul-smelling amniotic fluid were also more likely to be given ceftriaxone ((aOR 2.08, 95% CI 1.59–2.74), (aOR 1.34, 95% CI 1.03–1.75), (aOR 2.11, 95% CI 1.04–1.75)) and (aOR 5.93, 95% CI 1.36–25.83), respectively). However, patients with PROM were more likely to be given ampicillin than ceftriaxone (aOR 2.73, 95% CI 2.12–3.53).

### 2.2. Adherence to Guidelines on Antibiotic Prophylaxis in Labor and Delivery 

Referring to the local guidelines on prophylactic antibiotic administration in delivery, of the 3657 patients who had deliveries, 2725 (74.5%) had at least one indication for prophylactic antibiotic administration, and 932 (25.5%) had no indication. Among the 2725 patients who had an indication for prophylactic antibiotics, 1654 (60.7%) received prophylactic antibiotics, while 1071 (39.3%) did not. Among the 932 mothers who had no indications for prophylactic antibiotics, 67 (7.2%) still received antibiotic prophylaxis. The overall adherence to prophylactic antibiotic use guidelines was achieved in 68.9% of all deliveries. However, over the years, the proportion of adherence significantly decreased, decreasing from 77.2% in 2016 to 71.2% in 2017 and to 60.1% in 2018 (*p* < 0.0001).

The number of indications for prophylactic use was also associated with guideline adherence (*p* < 0.001). The highest adherence was noted in patients who had three indications (93.2%) followed by zero indications (89.6%) and two indications (77.3%), and the lowest level of adherence was in patients with one indication (59.3%). 

Multiple logistic regression showed that clinicians were more likely to adhere to the guidelines when the patient had PROM (aOR 27.88, 95% CI 17.17–45.26), antepartum hemorrhage (aOR 194.81, 95% CI 11.46 to >999.99), and foul-smelling amniotic fluid (aOR 3.65, 95% CI 1.26–10.58). However, adherence was significantly lower in more recent years (both 2017 (aOR 0.57, 95% CI 0.45–0.74) and 2018 (aOR 0.39, 95% CI 0.31–0.50) compared to the year of 2016) with preterm deliveries (aOR 0.37, 95% CI 0.25–0.54), with forceps extraction compared to spontaneous vaginal delivery (aOR 0.41, 95% CI 0.19–0.87), with maternal fever (aOR 0.52, 95% CI 0.29–0.95), and with a maternal leukocyte count > 15,000/mm^3^ (aOR 0.23, 95% CI 0.18–0.28), and it was the lowest when the patient only had one indication for prophylactic antibiotics (aOR 0.36, 95% CI 0.24–0.54) (Table 3).

## 3. Discussion

This study demonstrated that the prevalence of prophylactic antibiotic use in all deliveries was 47.1%. The overall prevalence of prophylactic antibiotic use during delivery in the current study was higher than in higher-income countries. Stockholm et al., through the Danish Copenhagen Prospective Study on Asthma in Childhood (COPSAC_2010_) in Denmark, reported that prophylactic antibiotic use during delivery was 33%, similar to the prevalence in the USA and Canada (30% and 39%, respectively) [27,28,29]. Compared to other studies from low- to middle-income countries, the current findings on prevalence were higher than those reported from Ghana, at 28%, but were much lower than those found in another study at a tertiary level hospital in India, where 994 of 1077 (92.3%) deliveries during the 2008–2010 period presented with indications that required the prescription of prophylactic antibiotics during and after delivery [16,17]. The main difference between the study in India and the others is that the study site in India had not yet implemented a general policy on antibiotic prescription, thereby showing the need for specific, well-defined, and evidence-based antibiotic prescribing guidelines in healthcare institutions to reduce inappropriate antibiotic use. The present study site is a top referral hospital, and the moderately high use of prophylactic antibiotics may reflect the admission of more complicated cases that were referred from other hospitals.

More than 60% of the prophylactic antibiotic use was in C-section deliveries. We observed that despite the strong recommendation in the guidelines for prophylactic antibiotic use for C-section, the prevalence of the practice was nearly 65%, which was lower than expected. The main possible reason for this situation is that some patients who had C-section deliveries were already being treated with antibiotics for therapeutic purposes, such as for urinary tract infections or for intraamniotic infections. Therefore, the administered antibiotics were not considered prophylactic antibiotics and were beyond the scope of this study. Another possible explanation, although less likely, was the possibility of inadequate patient management documentation, including prophylactic antibiotic administration, in the medical record. Despite the significant improvement in medical data recording, inaccurate and incomplete medical records remain a worldwide problem [30,31,32,33].

Our study also showed a significant decrease in prophylactic antibiotic use, which decreased from 59.2% in 2016 to 38.1% in 2018. However, this decline was followed by a significant decrease in the rate of clinician adherence to the guidelines. Therefore, the reduction in prophylactic antibiotic use may reflect an increased rate of non-adherence. Even though guidelines are believed to represent the best evidence and judgments, they are not fixed, mandatory protocols for healthcare providers. The decision to follow a guideline is independently based on the healthcare provider’s clinical judgment. The local guidelines recommend prophylactic antibiotics in C-section deliveries, PROM, cases of antepartum hemorrhage related to placenta previa, a maternal intrapartum temperature ≥ 38 °C, a maternal leukocyte count >15,000/mm^3^, and preterm deliveries (gestational age < 37 weeks) [34]. The local guidelines were developed by an independent committee by adopting other guidelines published by national and international health professional associations and Cochrane reviews [10,23,34,35,36,37,38,39,40]. In addition, adjustments were also made based on local data and took into account some local expert opinions. C-sections and PROM are conditions that are also recommended for prophylactic antibiotics by the American College of Obstetricians and Gynecologists (ACOG), as supported by consistent scientific evidence [23]. Antepartum hemorrhage related to placenta previa was recommended for prophylactic antibiotics because of the possibility of causing maternal infection [39]. Although our study site does not routinely implement culture-based screening for GBS due to resource constraints, intrapartum antibiotic prophylaxis (IAP) is advocated in preterm deliveries and for intrapartum temperature ≥ 38 °C [10,34]. At our study site, the administration of IAP is not limited to the prevention of GBS infection but is also performed to prevent other possible pathogenic infection in newborns. Therefore, in line with the local guidelines from the hospital’s pediatric department and the World Health Organization’s recommendation for preventing neonatal sepsis, prophylactic antibiotics are also recommended for women with maternal leukocyte counts > 15,000/mm^3^ [35,41]. 

This study found that, among the conditions recommended for prophylactic antibiotic administration, only C-sections, antepartum hemorrhage, and PROM were persistently associated with an increased risk of prophylactic antibiotic use. Maternal fever and isolated increased leukocyte counts were not associated with an increased risk of prophylactic antibiotic use but were significantly associated with non-adherence. These latter conditions were mainly adopted in the guidelines as part of a risk-based approach for neonatal GBS infection prevention [10]. In some countries, the risk-based approach is no longer used and has been replaced by culture-based screening to determine antibiotic use [42,43]. Previous studies have shown that the incidence of neonatal sepsis due to GBS infection is low in most Asian countries; therefore, this may be the reason why clinicians at our study site did not consistently administer prophylactic antibiotics to mothers solely based on the presence of one of these risk factors [44,45,46]. The same observation was also found in preterm birth cases, but our study did not find an increased risk of prophylactic antibiotic use, and clinician adherence was significantly low. This may reflect the fact that some inconsistent evidence still exists regarding the benefits of prophylactic antibiotics. Multiple clinical trials did not support routine prophylactic antibiotic administration to women in preterm labor with intact membranes in the absence of overt signs of infection. However, some studies have suggested that prophylactic antibiotics may prevent preterm births [23,47,48]. 

This study found that cefazoline, the type of antibiotic recommended by the guidelines for C-sections, was rarely used. Ceftriaxone was one of the most used antibiotics for prophylaxis in the current study setting. Drug sustainability may explain the choice of ceftriaxone compared to cefazoline in the pharmacy, as the supply of the former tends to be more stable and affordable. Ceftriaxone was also more commonly used due to common knowledge among practitioners that this antibiotic has excellent bioavailability and effectiveness, a low toxicity profile, and a long half-life [49].

This study showed that the healthcare providers had only moderate adherence to the guidelines, at 68%, regarding conditions indicated for prophylactic antibiotic. In general medical practices, a wide variation exists in terms of the guideline adherence rate, from 20% to nearly 100%. This rate varies according to the purpose of the guidelines, the definition of adherence employed, and the location of the study [50,51]. As expected, in our study, the proportion of adherence was higher for cases that had multiple conditions recommended by the guidelines for prophylactic antibiotic administration. This arguably shows that the health practitioners’ clinical judgment had a significant role in the decision-making process if the patient had only one risk factor for infection.

Given that the antibiotic prescribing guideline was formulated to minimize the emergence of antibiotic resistance and optimized patient treatment, the hospital’s stakeholders need to review the guideline implementation on a regular basis. Local healthcare providers should be involved in guideline development and review processes. In settings where guidelines are not strictly followed, factors that determine the drug prescription in developing countries include economic incentives, stable supplies, fear of unfavorable outcomes, peer norms, and local medical culture [52]. Consultation with targeted physicians would improve guideline adherence [53]. 

A significant association was observed between prophylactic antibiotic use and maternal education level, birth attendant, and history of multiple abortions. Our study had a high proportion of high school graduates, which may have lessened the power of our estimation; however, our study indicated that prophylactic antibiotics were given more frequently to high school graduates than to college graduates. A study by Stokholm et al. also found that education level was related to antibiotic use during pregnancy [27]. Higher education levels may enhance mother’s capacity to obtain and understand the importance of prenatal care and to receive important reproductive health services. In addition, a higher education level may indicate a better general health status or may influence the healthcare-seeking behavior of certain groups. This study found that this prophylactic antibiotic association was limited to high school graduate mothers and was not apparent in women with lower education levels. However, the collected data did not allow analysis of whether those with lower educational levels were receiving antibiotics for purposes other than prophylaxis. 

In this study, women with deliveries assisted by Ob/Gyns specialists were more likely to be given prophylactic antibiotics than women whose deliveries were assisted by residents. As the study site is a teaching hospital, most of the deliveries are assisted by a resident under the supervision of an Ob/Gyn specialist. If the delivery is too complicated and beyond the resident’s medical competency, the Ob/Gyn specialist then directly assists in the delivery. Therefore, this may explain the finding that Ob/Gyn specialists used more prophylactic antibiotics compared to residents. Previous studies have suggested that a physician’s experience may influence adherence to guidelines [54,55], but our study did not reveal this association. In a teaching hospital, residents rarely make independent decisions about patient treatment, so the specialists were more likely to be influencing prescription choices. 

Our findings suggest an association between a history of multiple abortions and prophylactic use. Pregnancy with a history of multiple abortions is frequently considered as a high-risk pregnancy due to its association with a higher risk of preterm birth, C-section deliveries, post-partum hemorrhage, PROM, and congenital malformation [56,57,58]; however, not enough evidence supports the need for prophylactic antibiotics in deliveries with a history of multiple abortions and no other signs of infection. Further studies may be needed to evaluate this association. 

This study had several study limitations. The first limitation was that the data were obtained by reviewing medical records, and the documentation may have been incomplete, thereby leading to some information bias. However, we were able to minimize information bias given that most of the collected data in the medical records were complete. In addition, this study involved data collection on a large sample and covered over 90% of the total deliveries in the study period; therefore, it arguably represents the study site’s overall situation. A second limitation was the retrospective nature of the study, as this precluded any further elaboration on the association of the clinician’s characteristics regarding adherence. However, the main objective of this study was to describe the practice of prophylactic antibiotic use in general and not to focus on clinician adherence. A further limitation was that the results of this study reflect the conditions in a single center in Indonesia and may not be generalizable. However, considering the large amount of data that we were able to collect from three consecutive years and given the fact that our study site is a large hospital that serves all primary, secondary, tertiary care from multiple neighboring cities, to some extent, our study could represent other teaching and public hospitals in Indonesia. However, it may not represent other private hospitals that mostly serve the wealthier proportion of the Indonesian population. The prophylactic antibiotic practices between teaching and private hospitals may differ substantially.

## 4. Materials and Methods

### 4.1. Study Design and Study Population

The study data comprised medical records from Mohammad Hoesin Hospital from 1 January 2016 to 31 December 2018. The hospital is a government-run teaching hospital and serves as the tertiary-level referral hospital for patients from five neighboring provinces. The hospital provides all primary, secondary, and tertiary care and has a near 1000-bed capacity. We identified all deliveries in this hospital based on the International Classification of Disease, 10th Revision, Clinical Modification (ICD-10-CM) codes (i.e., O60, O80–O84) and consulted the paper-based medical records of each patient. The hospital’s electronic medical record database indicated that 3957 deliveries had occurred during this period. We were able to collect complete data from 3657 medical records (91.5%), while 338 (8.5%) of the paper-based medical records were either lost or had missing sheets and were not included in the data analysis. 

### 4.2. Variables and Measurement 

The variable of interest in this study was prophylactic antibiotic use during deliveries. We determined that a prophylactic antibiotic was given when written and when specified as a prophylactic antibiotic in the medical record by the physician in charge. We recorded the maternal conditions that were recommended by the local guidelines for the administration of prophylactic antibiotics. According to the local guidelines, all C-section deliveries (elective and emergency), PROM, and cases with antepartum hemorrhage due to placenta previa were recommended for prophylactic antibiotics [34,36]. In addition, the guideline recommended for giving prophylactic antibiotics to mothers who had risk factors for infection, which included a maternal intrapartum temperature ≥ 38 °C, preterm deliveries (gestational age < 37 weeks), and a maternal leukocyte count > 15,000/mm^3^ [34]. The type of antibiotic was also recorded. 

We also documented other sociodemographic and obstetric variables, such as the mother’s age, place of residence, level of education, birth attendant, multiple births, gravidities, parities, number of abortions, and foul-smelling amniotic fluid. 

### 4.3. Statistical Analysis

The prevalence of prophylactic antibiotic use during deliveries from 1 January 2016 to 31 December 2018 was reported on a monthly basis. Poisson regression was used to assess the proportion of the use over the study period. Descriptive characteristics were summarized as frequencies and proportions. Comparisons between groups were assessed with the chi-square test. Logistic regression was used to assess the association of potential risk factors of antibiotic use and clinician adherence. Initially, each factor was tested individually in a univariate regression model. The variables with a *p*-value < 0.20 and the conditions that were recommended for antibiotic prophylaxis by the guidelines were then included in the final model to estimate the adjusted odds ratio. We derived the estimates (crude and adjusted odds ratio) with the corresponding 95% confidence intervals. A significance level of 0.05 was used in all of the analyses. The data processing and analyses were conducted using SAS^®^, version 9.4.

## 5. Conclusions

Our study demonstrated a moderate to high prevalence of prophylactic antibiotic use in our hospital. Maternal education level, the birth attendant, multiple abortions, C-sections, PROM, and antepartum hemorrhage were associated with prophylactic antibiotic use. Individual clinical judgment plays a vital role in the decision for prophylactic use and may lead to a low rate of clinician adherence to antibiotic prescribing guidelines. Therefore, clinicians, local stakeholders, and policymakers should be actively involved to ensure the development of guidelines that are based on the best and most recent scientific evidence and incorporate local data to ensure successful guideline implementation. 

## Figures and Tables

**Table 1:** Maternal sociodemographic and obstetric characteristics across study period.

Variables	2016	2017	2018	Total	*p* ^a^
	*n* = 1087	*n* = 1227	*n* = 1343	*n* = 3657	
**Maternal age group**					
<17 years old	5 (0.5)	6 (0.5)	12(0.9)	23 (0.6)	0.21
17–35 years old	851 (78.3)	979 (79.8)	1027 (76.5)	2857 (78.1)	
>35 years old	231 (21.3)	242 (19.7)	304 (22.6)	777 (21.3)	
**Mother Education**					
No formal education	3 (0.3)	23 (1.9)	7 (0.5)	33 (0.9)	<0.0001
Less than high school	72 (6.7)	120 (9.8)	193 (14.4)	385 (10.5)	
High school graduate	895 (83.2)	898 (73.2)	871 (64.9)	2664 (72.9)	
College or higher	106 (9.8)	130 (10.6)	192 (14.3)	428 (11.7)	
Missing ^b^	11 (1.0)	56 (4.6)	80 (5.9)	147 (4.0)	
**Mother’s place of residence**					
Resident	693 (63.8)	770 (62.6)	870 (64.8)	2333 (63.8)	0.54
Non-resident	393 (36.2)	456 (37.2)	470 (35.0)	1319 (36.1)	
Missing ^b^	1(0.1)	1 (0.1)	3 (0.2)	5 (0.1)	
**Birth attendant**					
Ob/Gyn	193 (17.8)	177 (14.4)	172 (12.8)	542 (14.8)	0.003
Resident	894 (82.2)	1050 (85.6)	1171 (87.2)	3115 (85.2)	
***Obstetric factors***					
**Parity ≥ 5**	15 (1.4)	30 (2.4)	25 (1.9)	70 (1.9)	0.17
**Previous abortion > 1**	34 (3.1)	43 (3.5)	23 (1.7)	100 (2.7)	0.01
**Gestational age < 37 weeks**	297 (27.3)	239 (19.5)	300 (22.3)	836 (22.9)	<0.0001
**Multiple birth**	33 (3.0)	21 (1.7)	32 (2.4)	86 (2.4)	0.11
**Mode of delivery**					
C-section	486 (44.7)	491 (40.02)	658 (49.0)	1635 (44.7)	0.0004
Spontaneous vaginal delivery	575 (52.9)	699 (57.0)	648 (48.3)	1922 (52.6)	
Vacuum extraction	11 (1.0)	14 (1.1)	21 (1.6)	46 (1.3)	
Forceps extraction	15 (1.4)	23 (1.9)	16 (1.2)	54 (1.5)	
**Intrapartum temperature ≥ 38 °C**	20 (1.8)	17 (1.4)	67 (5.0)	104 (2.8)	<0.0001
**PROM**	374 (34.4)	349 (28.4)	312 (23.2)	1035 (28.3)	<0.0001
**Maternal leukocyte count**					
>15,000/mm^3^	316 (29.1)	333 (27.1)	354 (21.2)	1003 (27.4)	0.25
<15,000/mm^3^	674 (62.0)	753 (61.4)	852 (58.3)	2279 (62.3)	
Missing ^b^	97 (8.9)	141 (11.5)	137 (20.6)	375 (10.3)	
**Antepartum hemorrhage**	22 (2.0)	24 (2.0)	16 (1.2)	62 (1.7)	0.2
**Foul-smelling amnion fluid**	7 (0.6)	17 (1.4)	11 (0.8)	35 (1.0)	0.15

^a^ *p*-values from chi-square tests; ^b^ Missing category were not included in the analysis.

**Table 2:** Association between prophylactic antibiotic use and maternal sociodemographic and obstetric factors.

Variables	Antibiotic Prophylaxis		OR (95% CI)	*p* ^a^	aOR (95%CI) ^†^	*p* ^a^
	Yes	No				
	(*n* = 1721)	(*n* = 1936)				
**Maternal age group**						
<17 years old	9 (0.5)	14 (0.7)	0.75 (0.32–1.72)	0.13	1.04 (0.32–3.35)	0.34
17–35 years old	1323 (76.9)	1534 (79.2)	Ref		Ref	
>35 years old	389 (22.6)	388 (20.0)	1.16 (1.00–1.36)		1.17 (0.94–1.48)	
**Mother Education ^b^**						
No formal education	13 (0.8)	20 (1.1)	0.84 (0.41–1.75)	0.009	0.83 (0.30–2.36)	0.01
Less than high school	159 (9.6)	226 (12.2)	0.92 (0.68–1.22)		0.91 (0.61–1.37)	
High school graduate	1298 (78.5)	1366 (73.6)	1.23 (0.99–1.54)		1.39 (1.04–1.86)	
College or higher	184 (11.1)	244 (13.2)	Ref		Ref	
**Mother’s place of residence ^c^**						
Resident	1055 (61.3)	654 (33.9)	Ref	0.003	Ref	0.08
Non-resident	666 (38.7)	1278 (66.2)	1.23 (1.08–1.41)		1.19 (0.98–1.45)	
**Birth attendant**						
Ob/Gyn	298 (17.3)	244 (12.6)	1.45 (1.21–1.75)	<0.0001	1.29 (1.001–1.66)	0.049
Resident	1423 (82.7)	1692 (87.4)	Ref			
**Parity**						
<5	1691 (98.3)	1896 (98.0)	Ref	0.48	--	--
≥5	30 (1.7)	40 (2.1)	0.84 (0.52–1.36)			
**Previous abortion**						
≤1	1663 (96.6)	1894 (97.8)	Ref	0.03	Ref	0.02
>1	58 (3.4)	42 (2.2)	1.57 (1.05–2.35)		1.88 (1.11–3.17)	
**Gestational age < 37 weeks**	450 (26.2)	384 (19.9)	1.42 (1.21–1.66)	<0.0001	1.13 (0.89–1.41)	0.31
**Multiple birth**	41 (2.4)	45 (2.3)	1.03 (0.69–1.57)	0.91	--	--
**Mode of delivery**						
C-section	1040 (60.4)	595 (30.7)	3.45 (3.01–3.96)	<0.0001	7.96 (6.40–9.91)	<0.0001
Spontaneous vaginal delivery	646 (37.5)	1276 (65.9)	Ref		Ref	
Vacuum extraction	16 (0.9)	30 (1.6)	1.05 (0.57–1.95)		1.54 (0.60–3.92)	
Forceps extraction	19 (1.1)	35 (1.8)	1.07 (0.61–1.89)		2.21 (1.00–4.85)	
**Intrapartum temperature ≥ 38 °C**	48 (2.8)	56 (2.9)	0.96 (0.65–1.42)	0.85	0.71 (0.43–1.17)	0.18
**PROM**	991 (57.6)	44 (2.3)	58.37 (42.65–79.89)	<0.0001	117.78 (83.28–166.58)	<0.0001
**Maternal leukocyte count**	474 (28.0) ^d^	528 (28.1) ^e^	0.99 (0.84–1.17)	0.93	--	--
**>15,000/mm^3^**						
**Antepartum hemorrhage**	62 (3.6)	0 (0)	145.87 (8.82–>999)	0.0005	309.93 (18.17–>999)	<0.0001
**Foul-smelling amnion fluid**	17 (1.0)	18 (0.9)	1.06 (0.54–2.07)	0.86	--	--

^a^ *p*-values from logistic regressions; ^†^ Adjusted for maternal age, education, place of residence, birth attendant, previous abortion, gestational age, mode of delivery, PROM, intrapartum temperature, maternal leukocyte count, antepartum hemorrhage, foul-smelling amniotic fluid; ^b^ missing 167; ^c^ missing 5; ^d^ missing 118; ^e^ missing 257.

**Table 3:** Adherence to local prophylactic antibiotic prescribing guidelines.

Variables	Adherence	Non-Adherence	OR (95% CI)	*p* ^a^	aOR (95% CI) ^†^
	(*n* = 2519)	(*n* = 1138)			
**Year of admission**					
2016	839 (33.3)	248 (21.8)	Ref	<0.0001	Ref
2017	873 (34.7)	354 (31.1)	0.73 (0.60–0.88)		0.57 (0.45–0.74)
2018	807 (32.0)	536 (47.1)	0.45 (0.37–0.55)		0.39 (0.31–0.50)
**Maternal age group**					
<17 years old	13 (0.5)	10 (0.9)	0.58 (0.25–1.33)	0.39	--
17–35 years old	1976 (78.4)	881 (77.4)	Ref		
>35 years old	530 (21.1)	247 (21.7)	0.96 (0.81–1.14)		
**Mother Education ^b^**					
No formal education	23 (1.0)	10 (0.9)	1.15 (0.54–2.49)	0.04	1.04 (0.34–3.11)
Less than high school	244 (10.1)	141 (12.9)	0.87 (0.65–1.16)		0.94 (0.63–1.39)
High school graduate	1867 (77.2)	796 (73.0)	1.18 (0.95–1.46)		1.19 (0.89–1.58)
College or higher	285 (11.8)	143 (13.1)	Ref		Ref
**Mother’s place of residence ^c^**					
Resident	1628 (64.8)	705 (62.0)	Ref	0.1	0.95 (0.78–112)
Non-resident	886 (35.2)	433 (38.0)	0.89 (0.77–1.02)		Ref
**Birth attendant**					
Ob/Gyn	375 (14.9)	167 (14.7)	1.02 (0.84–1.24)	0.87	--
Resident	2144 (85.1)	971 (85.3)	Ref		
**Parity**					
<5	2467 (98.1)	1116 (98.1)	Ref	0.95	--
≥5	48 (1.9)	22 (1.9)	0.99 (0.59–1.64)		
**Previous abortion**					
≤1	2449 (97.2)	1082 (97.4)	Ref	0.81	--
>1	70 (2.8)	30 (2.6)	1.06 (0.68–1.63)		
**Gestational age < 37 weeks**	450 (17.9)	386 (33.9)	0.42 (0.36–0.50)	<0.0001	0.37 (0.25–0.54)
**Multiple birth**	52 (2.1)	33 (3.5)	0.68 (0.44–1.06)	0.09	1.66 (0.90–3.03)
**Mode of delivery**					
C-section	1040 (41.3)	595 (52.3)	0.63 (0.54–0.72)	<0.0001	1.34 (0.09–2.00)
Spontaneous vaginal delivery	1414 (56.1)	508 (44.6)	Ref		Ref
Vacuum extraction	33 (1.3)	13 (1.1)	0.91 (0.48–1.75)		0.88 (0.37–2.05)
Forceps extraction	32 (1.3)	22 (1.9)	0.52 (0.30–0.91)		0.41 (0.19–0.87)
**Intrapartum temperature ≥ 38 °C**	48 (1.9)	56 (4.9)	0.38 (0.25–0.56)	<0.0001	0.52 (0.29–0.95)
**PROM**	991 (39.3)	44 (3.9)	16.13 (11.81–22.03)	<0.0001	27.88 (17.17–45.26)
**Maternal leukocyte count > 15,000/mm^3^**	549 (20.9) ^d^	482 (28.0) ^e^	0.34 (0.29–0.41)	<0.0001	0.23 (0.18–0.28)
**Antepartum hemorrhage**	62 (2.5)	0 (0)	3.31 (1.42–7.70)	0.006	194.81 (11.46–>999.99)
**Foul-smelling amnion fluid**	28 (1.0)	7 (0.6)	57.92 (3.50–958.43)	0.005	3.65 (1.26–10.58)
**Numbers of indications**					
**for antibiotic prophylaxis in a patient**					
None	873 (34.7)	312 (33.3)	Ref	<0.0001	Ref
One indication	924 (36.7)	638 (56.1)	0.52 (0.44–0.61)		0.36 (0.24–0.54)
Two indications	612 (24.3)	180 (15.8)	1.22 (0.98–1.50)		0.64 (0.31–1.29)
Three or more indications	110 (4.4)	8 (0.7)	4.91 (2.37–10.19)		0.63 (0.17–2.29)

^a^ *p*-values from logistic regressions; ^†^ Adjusted for admission year, maternal education, place of residence, multiple births, gestational age, mode of delivery, PROM, intrapartum temperature, maternal leukocyte count, antepartum hemorrhage, foul-smelling amniotic fluid, number of indications for antibiotic prophylaxis; ^b^ missing 167; ^c^ missing 5; ^d^ missing 266; ^e^ missing 109.

## Data Availability

All data generated and analyzed during this study have been included in this article.
